# Prescription of psychotropic medication in patients with type two diabetes mellitus: A multi-practice study from Ireland

**DOI:** 10.1080/13814788.2019.1640208

**Published:** 2019-07-23

**Authors:** Paul Keating, Ray O’Connor, Jane O’Doherty, Ailish Hannigan, Walter Cullen, Louise Hickey, Anne Harnett, David Meagher, Andrew O’Regan

**Affiliations:** a Department of General Practice, Graduate Entry Medical School, University of Limerick, Limerick, Ireland;; b Biostatistics at Graduate Entry Medical School, University of Limerick, Limerick, Ireland;; c School of Medicine, Health Sciences, UCD, Dublin, Ireland;; d Trinity College Dublin, Dublin, Ireland;; e Pharmacology at Graduate Entry Medical School, University of Limerick, Limerick, Ireland;; f Psychiatry at Graduate Entry Medical School, University of Limerick, Limerick, Ireland;; g General Practice at Graduate Entry Medical School, University of Limerick, Limerick, Ireland

**Keywords:** Antidepressants, benzodiazepines, healthcare utilization, primary care, type two diabetes mellitus

## Abstract

**Background:** Comorbid anxiety and depression and type two diabetes mellitus (T2DM) are commonly managed by General Practitioners (GPs).

**Objectives**: To investigate the proportion of people with T2DM who are prescribed either antidepressant or benzodiazepine medications in general practice; to compare people with T2DM that have a prescription with those that do not in terms of patient characteristics, glycaemic control and healthcare utilization.

**Methods**: Anonymized data was collected by GPs and senior medical students from electronic medical records of patients with T2DM in 34 Irish general practices affiliated with the University of Limerick Graduate Entry Medical School during the 2013/14 academic year. Data included demographics, healthcare utilization, prescriptions and most recent glycosylated haemoglobin (HbA1c) measurement.

**Results**: The sample included 2696 patients with T2DM, of which 733 (36.7%) were female, and with a median age of 66 years. The percentage with a current prescription for an antidepressant or benzodiazepine was 22% (95%CI: 18.9–24.9). Those with a current prescription for either drug were more likely to have attended the emergency department (28.3% vs 15.7%, *P* <0.001), to have been admitted to hospital (35.4% vs 21.3%, *P* <0.001) in the past year and attend their GP more frequently (median of 9 vs 7, *P* <0.001) than those without a prescription. Rates of poor glycaemic control were similar in those with and without a current prescription.

**Conclusion**: Over one-fifth of people with T2DM in Irish general practice are prescribed an antidepressant or benzodiazepine medication. Prescription of these is associated with increased healthcare utilization but not poorer glycaemic control.

KEY MESSAGES

- Over one in five patients with T2DM in general practice had a current prescription for antidepressants or benzodiazepines
- These patients have higher primary and secondary healthcare utilization
- There is no difference in rates of glycaemic control for patients with or without a current prescription for an antidepressant or a benzodiazepine

## Introduction

Depression and anxiety frequently co-exist in the general population and commonly occur in people with diabetes mellitus with the presence of one increasing the risk of developing the other [1,2]. Furthermore, the comorbid state is associated with poorer outcomes for each condition [3]. Depression occurring in the type two diabetes mellitus (T2DM) population tends to have a particularly strong anxiety component [4]. General practitioners (GPs) are responsible for diagnosing and treating most people with T2DM and comorbid depression and anxiety, frequently prescribing benzodiazepine and antidepressant medications [5,6]. As well as their use in the management of depression and anxiety [7], these medications are commonly prescribed to people with untreated and undiagnosed depression [8]. Both classes of medication are associated with significant physical health risks [9,10].

Depression in T2DM has a point prevalence of 10–15% but there is no data on the proportion of people with T2DM that are prescribed an antidepressant or a benzodiazepine medication [11]. When either depression or anxiety, or both, are present in people with diabetes, the risk of mortality increases [12]. The reasons for this are unclear but it is an important clinical point for GPs who manage these conditions daily. Similarly, the effect of comorbid depression and anxiety on glycaemic control remains uncertain [13]. Screening for depression and anxiety is not routinely recommended in the general practice care of people with T2DM, but knowing the patient characteristics that are associated with these conditions may help GPs in their management. Furthermore, research on a range of chronic illness reported that comorbid depression and anxiety symptoms were associated with twice the likelihood of health service use [14]. There is no available data on the effect of these symptoms on the health service use of people with T2DM in general practice. With GPs expected to coordinate secondary care referrals and appropriate use of service resources, knowledge on healthcare utilization among this population is important for health planning [15].

The objectives of this study, therefore, were first, to establish what proportion of people with T2DM were prescribed benzodiazepine or antidepressant medications, and second, to investigate the difference in patients prescribed these medications to those who were not with regard to:

1. Practice and patient characteristics
2. Glycaemic control, using glycosylated haemoglobin (HbA1c)
3. Health service utilization (of primary and secondary healthcare services)

## Methods

### Study design

This was an observational study involving a retrospective analysis of patient records. It is part of a larger study on the management of patients with T2DM in general practice [16].

### Ethics

The Mid-Western Regional Hospital Research Ethics Committee granted ethical approval for the study (22 May 2013).

### Study setting

All 51 practices affiliated with the University of Limerick Graduate Entry Medical School, located in the mid-west of Ireland, with a senior medical student on placement in 2013/14 were invited to participate in this study. These practices are based in three of Ireland’s four healthcare regions, which serve a population of 3.5 million. Ireland has a mixed public/private healthcare system, with approximately 43% of the population eligible for free healthcare [17]. At the time of data collection, there was no structured delivery of care nationally to patients with T2DM in primary care in Ireland.

### Study sample

Senior medical students on placement and their GP supervisors used existing diabetes registers or reporting functions of electronic practice management systems to generate a list of all patients with T2DM in the practice. Where no register existed, patients were identified using disease coding, medications or the recording of the use of blood glucose monitors in the practice management system. The practice team reviewed all lists to ensure they were up to date and complete. For feasibility of detailed data extraction and to ensure comparable workload for students on placement across practices, in practices with over 100 patients with T2DM, a random sample of 100 patients was selected using the random number function in Microsoft Excel.

### Measurements

A standardized data extraction tool was used to record the following information for those patients with T2DM who had consulted with their GP in the previous year (1 September 2012 to 31 August 2013):

- Age
- Gender
- Eligibility for free healthcare
- Current prescription for antidepressants or benzodiazepine
- Smoking status: current smoker, non-smoker or smoking status not recorded in the previous year
- Body mass index (BMI): BMI <25 kg/m2; BMI of 25–30 kg/m2; BMI ≥ 30 kg/m2 or BMI not recorded in the previous year
- Healthcare utilization in the previous year: total number of GP consultations where a consultation is defined as any visit or telephone conversation that resulted in an entry to patient records; any attendance to the emergency department; any hospital admission; any referral or attendance to specialist mental health services; any referral or attendance to an endocrinologist
- most recent measurement of HbA1c in mmol/mol. Poor glycaemic control was defined as HBA1c greater than 8% (64 mmol/mol)
- whether the practice was in an urban (concentrated geographical area with 5000 or more residents), rural (scattered population over a large geographical area) or mixed urban/rural location (both concentrated and scattered population) [18]
- a classification of the deprivation of the area in which the practice was located based on the All-Ireland HP Deprivation Index [19]
- the number of full-time GPs in the practice
- the number of part-time and full-time practice nurses
- whether the practice was a member of a primary care team

Data were entered using the standardized data template in each practice and anonymized data sets from all practices were merged into a master file.

### Statistical analyses

The proportion of patients with a current prescription for antidepressants or benzodiazepines was estimated with a 95%CI for the proportion, using the SAS procedure SURVEYFREQ to account for clustering of patients within practices. Demographic and healthcare utilization variables were summarized using graphical and numeric descriptive statistics. Medians were compared across groups (those with a current prescription, those without) using non-parametric tests and Pearson’s chi-squared test was used to investigate associations between categorical variables. A multiple logistic regression analysis was carried out to measure associations between current antidepressant or benzodiazepine prescription (yes/no) using both patient level characteristics and practice level characteristics as explanatory variables. A 5% level of significance was used for all tests and odds ratios with 95%CIs are reported.

## Results

### Practices

Out of the 51 practices were invited to participate in this study and a total of 34 practices participated (67% of eligible practices). Practice size ranged from 1169–22 352 patients with a median of 5160 patients. Most (79%) of participating practices had an existing diabetes register. All patients with T2DM were included from half of the participating practices with the numbers of less than 100, and, in the remaining half of practices, a random sample of 100 patients with T2DM was selected. Table 1 outlines the characteristics of participating practices (*n* = 34 practices).

**Table 1.** Characteristics of participating practices (*n* = 34 practices).

Practice characteristics	Practices *n* (%)
Number of full-time GPs	
One/two	17 (50)
Three/four	17 (50)
Practice nurse	
Full-time	20 (58.8)
Part-time	14 (41.2)
Location	
Urban	8 (23.5)
Rural	14 (41.2)
Mixed urban/rural	12 (35.3)
Member of a primary care team	11 (32.4)
Deprivation index	
Disadvantaged	10 (29.4)
Marginally below average	13 (38.2)
Marginally above average	8 (23.5)
Affluent	3 (8.8)

### Patients with and without a prescription for a psychotropic drug

The final sample was 2696 patients with T2DM who had visited their GP in the previous year. The median age of the sample was 67 years (range: 11–100 years) and the majority of the sample were male (61%) and eligible for free healthcare (77%). The percentage of patients with T2DM with a current prescription for an antidepressant or benzodiazepine was 22% (95%CI: 18.9–24.9).

Table 2 compares the characteristics and healthcare utilization of patients with and without a current prescription. Those with a current prescription for antidepressants or benzodiazepine were more likely to be female, eligible for free healthcare and be recorded as a current smoker compared to those without a current prescription (Table 2). Those with a current prescription were more likely to have attended the emergency department, be admitted to hospital, have been referred to or attended specialist mental health services in the past year and consult with their GP more frequently compared to those without a current prescription (Table 2). There were no differences in age, BMI or the rates of referral/attendance to an endocrinologist between the two groups.

**Table 2.** Characteristics and healthcare utilization of patients with type two diabetes mellitus, with or without a current prescription for antidepressants or benzodiazepine.

	Patients without a current prescription (*n* = 2106)	Patients with a current prescription (*n* = 590)	*P*-value
Age			
Median (range)	66 years (11–100 years)	67 years (27–96 years)	0.22
Female gender	733 (36.7%)	287 (48.6%)	<0.001
Eligible for free healthcare	1566 (74.5%)	519 (88.1%)	<0.001
Current smoking status			
Unknown	1015 (48.2%)	286 (48.5%)	<0.001
Non-smoker	868 (41.2%)	209 (35.4%)	
Current smoker	223 (10.6%)	95 (16.1%)	
Body mass index (BMI)			
BMI not recorded	1056 (50.2%)	305 (51.7%)	0.13
BMI <25 kg/m^2^	129 (6.1%)	31 (5.3%)	
BMI 25–30 kg/m^2^	386 (18.3%)	87 (14.7%)	
BMI ≥ 30 kg/m^2^	535 (25.4%)	167 (28.3%)	
HbA1c			
Median	6.8% (51 mmol/mol)	6.8% (51 mol/mol)	
First to third quartile	6.3–7.6%	6.3–7.7%	
Exceeds cut-off point of 8% (64 mmol/ mol)	18.2%	18.9%	0.71
Number of GP consultations			
Median	7	9	<0.001
First to third quartile	3–12	5–16	
Secondary healthcare utilization			
Attendance at the emergency department	331 (15.7%)	167 (28.3%)	<0.001
Referral/attendance to endocrinologist	447 (21.2%)	110 (18.6%)	0.17
Referral/attendance to specialist mental health services	24 (1.1%)	128 (21.7%)	<0.001
Hospital admission	448 (21.3%)	209 (35.4%)	<0.001

### The relationship between glycaemic control and psychotropic drug use

A recent measurement (within the previous 12 months) of HbA1c was available for 88% of the patients (*n* = 2362). The distributions of the most recent measurement of HbA1c were similar for both groups (Table 2).

Using a cut-off of greater than 8% (64 mmol/mol) to represent poor glycaemic control, a similar proportion (of approximately 18.3%) in both groups were above the cut-off point.

### Factors associated with prescription of psychotropic drugs

A multiple logistic regression analysis to measure associations between practice and patient characteristics with having a current prescription for antidepressants or benzodiazepines identified that female gender, being eligible for free healthcare, being a current smoker and consulting with the GP more frequently were independently associated with statistically significant increased odds of having a current prescription (Table 3). An urban location for the practice was also associated with statistically significant increased odds of having a current prescription (Table 3).

**Table 3.** Multiple logistic regression of current antidepressant or benzodiazepine prescription (yes, no) in patients with type two diabetes mellitus (*n* = 2,696)

Explanatory variable^a^	Odds ratio (95%CI)	*P*-value
*Patient characteristics*		
Gender		
Female gender (male = 1)	1.43 (1.16–1.78)	0.0009
Eligible for free healthcare (no = 1)	2.17 (1.62–2.90)	<0.0001
Number of GP consultations	1.04 (1.02–1.05)	<0.0001
Smoking status (non-smoker = 1)		
Smoker	1.74 (1.26–2.40)	0.0014
Unknown	1.24 (0.92–1.67)	0.64
*Practice characteristics*		
Practice location (mixed = 1)		
Urban	1.55 (1.11–2.16)	0.0005
Rural	0.86 (0.56–1.32)	0.052

a Final model presented with all variables statistically significant (*P* <0.05).

## Discussion

### Main findings

This study, conducted in 34 practices in Ireland, including 2696 patients with T2DM who had visited their GP in the previous year, found 22% of these patients had a prescription for an antidepressant or benzodiazepine. Those with a current prescription were more likely to have attended the emergency department, be admitted to hospital, have attended specialist mental health services in the past year or have attended the GP more frequently compared to those without a prescription. There was no significant difference between rates of glycaemic control for those with or without a current prescription for antidepressants or benzodiazepines. In a multivariable model, we identified five independent factors associated with the prescription of a psychotropic drug in this population: female gender, frequent attendance to the GP, tobacco smoking, attendance at urban centres and eligibility for free healthcare.

### Prevalence of psychotropic drug prescription

The figure of 22% of the T2DM population in this study prescribed these drugs may be explained by previous findings that up to 20% of people with any type of diabetes mellitus have a major depressive disorder [20].

A recent study of benzodiazepine prescribing showed that the rate of prescribing has been decreasing in Ireland [21]. The paper reported that in 2015 the rate of prescribing of benzodiazepines in the general population was 16.6% [21]. Similarly, antidepressant prescribing rates vary between countries, from 3% in Dutch centres to 9% in UK centres [22]. The most recent available analysis in Ireland reports an antidepressant prescribing rate of 5% [23]. In broad terms, the combined prescribing rates in the general population do not appear to be dissimilar to the prescribing rate of people with T2DM reported in this study. However, a crude combination would likely overestimate the actual numbers prescribed these medications as it would twice include the significant number of people likely be prescribed both drugs.

### Psychotropic drugs and glycaemic control

Our finding that glycaemic control, as measured by HBA1c, is not significantly associated with antidepressant or benzodiazepine use, contributes new information to an important clinical question. The relationship between mood and glycaemic control is unclear, with one study finding that depressive symptoms were not associated with poor glycaemic control in T2DM [24], while another study showed a sustained improvement in mood was associated with improved glycaemic control in T2DM [25]. In this study, a less stringent cut-off point was applied because of the presence of older age profile and presence of comorbidities [26]. This may lead to a comparatively lower proportion of people with ‘poor’ glycaemic control.

### Psychotropic drugs and healthcare utilization

While people that are prescribed antidepressants or benzodiazepines had similar glycaemic control to those that did not, they were more likely to attend the GP as well as secondary care services. The fact that an underlying psychological condition is expected to be present may explain the higher attendance to specialist mental health services, but the study also found higher attendances at the emergency department as well as being more likely to be admitted to hospital. Interestingly, the rates of attendance at specialist endocrinology services were similar, suggesting that there may not necessarily be a higher rate of diabetes-related complications among people prescribed these medications. The presence of comorbid major depression in any physical condition is linked to approximately double the likelihood of healthcare utilization [14]. High-risk health behaviour, including smoking, and increased incidence of adverse heart events have been reported in people with depression in T2DM [27] and this may account for the increased use of emergency departments and inpatient admissions. The increased secondary care utilization has health service planning implications as management of T2DM in primary care is much more cost-effective [28] and people with the condition who are managed in primary care have better indices of quality of life with similar health outcomes compared to secondary care [29].

### Factors associated with prescription of psychotropic drugs

The association we found with female gender could be explained by research showing that women with a physical illness were more likely to access mental health services than men and that for women more than men with depressive symptoms [30], the risk of developing T2DM is higher [31]. The association with higher GP attendance rates is similar to findings reported by Brieler et al., and is possibly because the high prevalence of anxiety-related symptoms may increase these patients’ attendance rates [25,32]. We found no higher prescribing prevalence in people with T2DM in areas of social deprivation but prevalence was higher in people who were eligible for unrestricted public medical care (mostly lower income) similar to Jacob et al. [33]. The association with smoking and having a prescription for a psychotropic medication is consistent with previous research showing increased rates of smoking in people that are prescribed this medication [34].

### Strengths and limitations

The strengths of this study include the large sample size of clinical records of patients reviewed across a large number of practices and the scope of the examination of clinical records. The practices that participated are considered a representative sample of all practices by size, urban/rural location and patient eligibility for free care.

Study limitations include that we only can say that benzodiazepine or antidepressant drugs were or were not prescribed. We have used prescribing as a proxy for mental illness while acknowledging that a minority of prescriptions for these medications may be used for the treatment of neuropathic pain or sleep disorders. The observational design of this study limits the strength of the conclusions we can draw about the differences between those with a current prescription or not. We also have not compared our results on patients with T2DM to patients without T2DM who are currently prescribed antidepressants or benzodiazepines. No exact dates of measurement for HbA1c were collected but all measurements were taken within the previous 12 months. Lastly, objective glucose measurements, with which to make comparisons, were not available.

### Further research

The authors recommend that future research should focus on the reasons for increased secondary healthcare use in patients with a current prescription and the appropriateness of this use, particularly of the emergency department.

## Conclusion

This study highlights that, among a T2DM general practice population, over one in five are prescribed antidepressant and benzodiazepine medication. Having a prescription for these medications is associated with increased secondary healthcare use, in particular attendance at emergency departments and hospital admission, which has serious implications for individual health, quality of life and health service planning.
